# Tumor Suppressor WWOX inhibits osteosarcoma metastasis by modulating RUNX2 function

**DOI:** 10.1038/srep12959

**Published:** 2015-08-10

**Authors:** Sara Del Mare, Rami I. Aqeilan

**Affiliations:** 1 The Lautenberg Center for Immunology and Cancer Research, IMRIC, Faculty of Medicine, Hebrew University of Jerusalem, Israel 91220

## Abstract

Osteosarcoma (OS) is among the most frequently occurring primary bone tumors, primarily affecting adolescents and young adults. This malignant osteoid forming tumor is characterized by its metastatic potential, mainly to lungs. We recently demonstrated that WW domain-containing oxidoreductase (WWOX) is frequently inactivated in human OS and that WWOX restoration in WWOX-negative OS cells suppresses tumorigenicity. Of note, WWOX levels are reduced in paired OS samples of post-treatment metastastectomies as compared to pre-treatment biopsies suggesting that decreased WWOX levels are associated with a more aggressive phenotype at the metastatic site. Nevertheless, little is known about WWOX function in OS metastasis. Here, we investigated the role of tumor suppressor WWOX in suppressing pulmonary OS metastasis both *in vitro* and *in vivo*. We demonstrated that ectopic expression of WWOX in OS cells, HOS and LM-7, inhibits OS invasion and cell migration *in vitro*. Furthermore, WWOX expression reduced tumor burden *in vivo* and inhibited metastases’ seeding and colonization. Mechanistically, WWOX function is associated with reduced levels of RUNX2 metastatic target genes implicated in adhesion and motility. Our results suggest that WWOX plays a critical role in determining the aggressive phenotype of OS, and its expression could be an attractive therapeutic target to combat this devastating adolescent disease.

Osteosarcoma (OS) is an aggressive malignant tumor arising from primitive transformed cells that exhibit osteoblastic differentiation and produce malignant osteoid tissue^1^,^2^. Approximately 30% of patients with OS present clinically detectable metastatic disease at diagnosis^3^,^4^. For patients with metastatic disease at initial presentation, 20% will remain continuously free of disease, and roughly 30% will survive 5 years from diagnosis. Lung is the most common site of initial metastatic disease^4^. Patients with metastases limited to the lungs have a better outcome than patients with metastases to other sites or to the lungs combined with other site^5^. The metastatic process comprises a series of steps all of which requiring the participation of specific molecules/genes. In order to sustain its growth, the primary tumor starts to form new blood vessels. In addition, neovascularization provides the necessary network for the development of metastasis. Neoangiogenesis is followed by migration, invasion, anoikis resistance, extravasation and colonization in the distant organ^6^. Finding new key molecules and understanding their role in one or more of those steps is essential for the development of more effective therapies that specifically target OS metastatic cells.

Inactivation of tumor suppressors is a hallmark of cancer^7^, including OS. Several lines of evidence have indicated that the WW domain-containing oxidoreductase (WWOX) acts as a tumor suppressor (reviewed in^8^,^9^,^10^). WWOX loss is a common event in almost all cancer types due to hemizygous deletions in most cases but also homozygous deletions were reported^10^,^11^. We have previously shown that loss of function of tumor suppressor WWOX is associated with OS tumorigenesis both *in vivo* and *in vitro* and its expression is absent or reduced in the majority of OS cases^12^. In particular, we showed that WWOX expression, as assessed by immunohistochemistry, is often reduced or absent in primary OS samples but also in paired samples from patients who had a pre-treatment OS biopsy and a post-treatment metastastectomy^12^ suggesting that low levels of WWOX are associated with a more aggressive disease at the metastatic site. Other studies in human OS revealed frequent alterations of the *WWOX* gene by genome-wide array CGH analysis^13^, of *WWOX* mRNA by real-time PCR^14^ and of WWOX protein by immunohistochemical staining^13^. When WWOX loss is modeled in mice, *Wwox* null mice die prematurely at age of 3–4 weeks with metabolic and neurological disorders^15^,^16^,^17^,^18^. Careful investigation of *Wwox* mutant mice prior to their death revealed that WWOX possesses tumor suppressor functions^15^,^17^,^19^,^20^. In this respect, we demonstrated that loss of both alleles of *Wwox* results in lesions resembling OS in juvenile mice^12^,^15^,^17^ whereas *Wwox*-heterozygous mice develop spontaneous and chemically-induced tumors^15^,^19^,^21^. Altogether, these data suggest that WWOX is playing a critical role in OS development and functions as a tumor suppressor. Nevertheless, the role of WWOX in metastases, and in particular OS metastasis, is not well defined.

The current therapeutic strategies have a limited efficacy in the treatment of metastatic disease, and the metastatic relapse or recurrent conditions have remained unchanged over the last three decades^22^. Treating metastatic OS remains a serious challenge in bone cancer^23^ and finding new therapeutic targets to one or more of the metastatic stages is essential to improve OS therapy. Here, we investigated the role of tumor suppressor WWOX in OS metastasis and found that ectopic expression of WWOX attenuates migration and invasion *in vitro* and metastasis *in vivo* through inhibition of key metastatic genes implicated in OS.

## Materials and Methods

### Cell lines and cell culture

HOS cell line was obtained from the American Type Culture Collection. LM7 cell line was a gift of Dr. Eugenie Kleinerman (The University of Texas MD Anderson Cancer Center, USA). Cells were cultured in RPMI 1640 supplemented with 10% fetal bovine serum.

### *In vitro* viral transduction

To generate a lentiviral WWOX vector, human WWOX cDNA was cloned in lentiviral vector containing neomycin as the selection marker using the Gateway cloning system (Invitrogen, Grand Island, NY). Cells were transduced with Lenti-WWOX or Lenti-EV at appropriate multiplicities of infection. Cells were next labeled with green fluorescent protein (GFP) using Lenti-GFP particles and assessed by immunoblotting using anti-WWOX polyclonal antibody and visualization of GFP. Cells were put in selection with 2 μg/mg of neomycin.

### Wound healing assay

Cells were grown to confluence in a 6-well plate, and then scratched using a 200 μl sterile pipette tip. Images of wounded monolayers were photographed under an inverted microscope at the time of wounding (0 h), 9 and 24 hrs later. Quantification of the % of wound closure in WWOX-manipulated cells was done using the software ImageJ in three spots of the wound on each triplicate. Results were expressed as mean ± STDV.

### Matrigel invasion assay

Blind well chemotaxis chambers with 13-mm-diameter filters were used for this assay. Polyvinylpyrrolidone-free polycarbonate filters, 8-mm pore size (Costar Scientific Co., Cambridge, MA), were coated with basement membrane Matrigel (25 μg per filter). Briefly, the Matrigel was diluted to the desired final concentration with cold distilled water, applied to the filters, and dried under a hood. HOS or LM7 cells (2 × 10^5^), suspended in DMEM containing 0.1% bovine serum albumin, were added to the upper chamber. Conditioned medium of NIH3T3 fibroblasts was applied as a chemoattractant and placed in the lower compartment of the Boyden chamber. Assays were carried out at 37 °C in 5% CO_2_. Over 90% of the cells attached to the filter after incubation for 7 h. At the end of the incubation, the cells on the upper surface of the filter were removed by wiping with a cotton swab. Cells that passed trough the filter into the bottom side of the membrane were fixed with methanol and stained with Giemsa. Each assay was done in triplicate and ten representative fields in each well were quantified to determine the number of invasive cells under a light microscope.

### Intravenous and orthotopic intratibial injection

All animal work was performed in accordance with the guidelines of the Institutional Animal Care and Use Committee of The Hebrew University of Jerusalem under approved protocol number, MD-11–12856-5; NIH approval number: OPRR-A01-5011. Four to six week old male NOD/SCID mice were used for the experiment. WWOX manipulated HOS and LM7 GFP+ cell lines were allowed to reach subconfluency, harvested with Trypsin-EDTA (Life Technologies), and injected intravenously (i.v.) into the lateral tail vein of mice at a density of 1 × 10^6^ cells/0.2 ml serum-free medium. Mice were sacrificed 2 months after injection.

For intratibial injections, exponentially growing GFP-tagged OS cells were harvested, counted, and resuspended in PBS to a final concentration of 10^7 ^cells/ml. 1 × 10^6^ cells/0.1 ml serum-free medium were injected into the right proximal tibia. Briefly, the animals were anesthetized with ketamine (80 mg/kg) and xylazine (7 mg/kg). The knee of the NOD SCID mouse was flexed beyond 90^o^ and a 100 μl of the cell suspension was injected into the proximal tibia using a 25-gauge needle. Five animals were injected with each OS-manipulated cell line. Animals were monitored daily, and tumor sizes were measured every week. At the end point animals were sacrificed, tumors were weighed, and tumor volumes were calculated as previously described^12^. Animals were sacrificed at ~3 weeks (for HOS EV tumors) and 5 weeks (for HOS-WWOX tumors). The animals were euthanized in a carbon dioxide chamber, followed by necropsy. Metastatic foci (micro less than or equal to 2 mm; macro greater than 2 mm) were counted using fluorescent stereoscope.

### RNA extraction and quantitative RT-PCR

RNA was isolated from cell lines using TRIzol reagent (Invitrogen) according to the manufacturer’s protocol. cDNA was synthesized with oligo (dT) primers using the super-script first strand synthesis kit (Invitrogen) according to the manufacturer’s protocol. RealTime PCR was run on Applied Biosystem 7900HT machine using Sybr Green probe (ABI, Warrington WA1GSR, UK)) at standard conditions. The primers used for qRT-PCR used are summarized in the Table 1.

### Statistics

Results of *in vitro* and *in vivo* experiments were expressed as mean ± SD or SE. Fisher’s exact test and Student’s t test were used to compare values of test and control samples. P < 0.05 indicated significant difference.

## Results

### WWOX restoration in HOS and LM7 osteosarcoma cells results in attenuation of migration and invasion capabilities *in vitro*

**Figure 1:**
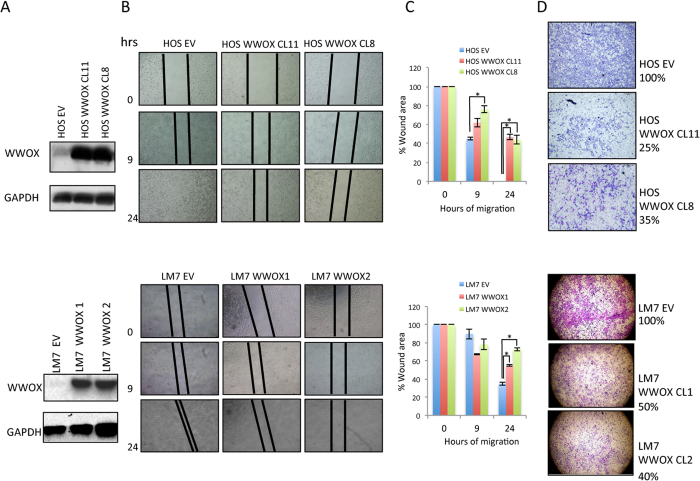
Restoration of WWOX in OS cells suppresses migration and invasion *in vitro*. (**A**) Western blot analysis of WWOX expression in HOS (upper panel) and LM7 (lower panel)- EV and WWOX cells showing restoration of WWOX. GAPDH served as loading control. Images shown were cropped to indicate relevant lanes. (**B**) Wound healing assay, reduced migration of HOS and LM7 WWOX-expressing compared to control cells. Wound closures were photographed at 0, 9 and 24 hrs after scratch. Cells were grown serum-free media. The scale lines represent scratch. (**C**) Quantification of the % of wound closure in WWOX-manipulated cell lines from (**B**). Wound size at different time points was measured using the software ImageJ in three spots of the wound on each triplicate. Results are expressed as mean ± SD. *p < 0.01 as compared with HOS/LM7-EV cells. (**D**) Matrigel invasion assay. HOS and LM7 stable *WWOX* clone exhibits markedly reduced Matrigel invasion properties (*P *= 0.003).

We previously demonstrated that WWOX levels are reduced in most OS cells^12^. Indeed, both HOS and LM-7 metastatic OS cells express very low or undetectable levels of endogenous WWOX (Fig. 1A). To study effect of WWOX expression on metastatic traits in these cells, we used lentiviral vectors to generate stable clones of HOS and LM7 cells expressing WWOX or empty vector (EV) and then measured their cell migration and invasion characteristics *in vitro*. Overexpression of WWOX was validated by immunoblotting using anti-WWOX antibody (Fig. 1 upper panel). To examine effect of WWOX on cell migration, we performed a wound-healing assay and found that HOS-WWOX cells have low cell motility compared with HOS-EV cells when grown in a serum-free media (Fig. 1B,C). Next, we determined WWOX action on invasion using a Boyden chamber assay and found that HOS-WWOX cells exhibited reduced invasiveness relative to EV-expressing cells (Fig. 1D). Similar traits were observed when LM7-WWOX stable clones were compared with LM7-EV cells (Fig. 1 lower panel). These data suggest that WWOX restoration can attenuate metastatic characteristics *in vitro* in metastatic OS cell lines.

### WWOX restoration in HOS cells inhibited their metastatic potential when injected intratibially in NOD-SCID mice

**Figure 2:**
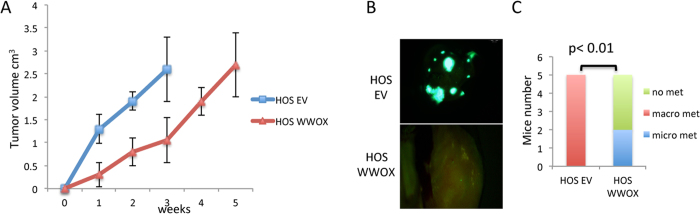
WWOX-overexpressing HOS cells display an impaired metastatic potential when injected intratibially. (**A**) HOS EV derived tumors grow much faster than HOS WWOX tumors. Measurements of tumor volumes were performed every week. (**B**,**C**) HOS-EV-GFP and HOS-WWOX-GFP cells were injected into the tibia (IT) of NOD/SCID mice (5 mice per group). Lung metastases were visualized by fluorescent imaging after 5 weeks from injection. (**B**) Representative images showing macrometastasis in HOS cells. (**C**) Quantification of micro (less than or equal to 2 mm) and macro (greater than 2 mm)–metastasis, as assessed by counting using fluorescent stereoscope.

The presence of metastasis at diagnosis is the most important predictor of disease-free survival with a 5-year survival rate of only 20% for OS patients with metastasis compared to 65% for patients with localized disease^4^. Since our findings indicated that WWOX significantly diminish migration and invasion potential of OS cells *in vitro*, we next set to examine whether WWOX expression inhibits seeding of metastasis *in vivo*. In order to assess whether WWOX could affect the metastatic potential of OS cell lines, we labeled manipulated HOS or LM-7 cells with GFP, injected them intratibially (IT) and followed formation of primary tumors and examined for lung metastasis, the predominate site of OS metastasis. By three weeks of IT injections, HOS EV cells formed large tumors that mice had to be removed from the experiment (Fig. 2A). By contrast, mice injected with WWOX-expressing HOS cells displayed significantly smaller tumors as compared to HOS-EV cells (0.5 cm^3^ compared to 2.0 cm^3^, respectively) (Fig. 2A) To compare metastatic lesions, HOS-WWOX injected mice were sacrificed at ~5-weeks allowing primary tumors to reach comparable size to HOS-EV bearing mice. As shown in Fig. 2B,C, higher incidence of pulmonary metastasis was observed in matched sized tumor bearing-mice inoculated with HOS-EV cells as compared to HOS-WWOX cells. In fact, all HOS-EV IT-injected mice developed macrometastasis whereas 2 out of 5 (40%) mice injected with HOS-WWOX developed only micrometastasis (Fig. 2C). These data suggest that WWOX overexpression leads to an attenuation of the metastatic seeding potential of HOS cells when injected orthotopically. LM-7 cells did not form tumors in the IT model in same time period and therefore WWOX effect on metastatsis using this model could not be assessed.

### WWOX restoration in OS cells inhibited their metastatic potential when injected intravenously in NOD-SCID mice

**Figure 3:**
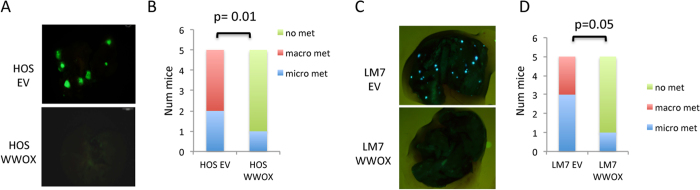
WWOX expression inhibited OS metastatic potential when injected intravenously. (**A,B**) HOS-EV-GFP and HOS-WWOX-GFP (**A,B**) and LM7 -EV-GFP and LM7-GFP-WWOX cells (**C,D**) were injected into the lateral tail vein (IV) of NOD/SCID mice (5 per group). The formation of metastasis in the lungs was detected after 8 weeks from the injection. Images obtained by fluorescent microscopy displaying macrometastasis in HOS and LM7 cells are shown. Quantification of micro and macro –metastasis (as in Fig. 2C) is shown in (**B,D**).

We next determined whether WWOX impact on invasion and metastasis was also attributable to effects on later steps of the invasion-metastasis cascade, independent of its influence on local invasion (IT model). Thus we injected WWOX-expressing HOS or LM-7 cells directly on the circulation of mice, thereby circumventing the initial steps of local invasion and intravasation. To this end, GFP-fluorescent OS cells were injected intravenously (IV) and formation of GFP+ metastatic lung nodules was assessed. Two months after tail-vain injection, WWOX-expressing HOS cells generated fewer lung metastases than did controls (Fig. 3A). In fact, all mice injected with HOS-EV developed macrometastasis (60%) and micrometastasis (40%) whereas only 1 out of 5 (20%) of mice bearing HOS-WWOX cells in the IV model develop micrometastasis (Fig. 3B). Similar results were obtained with the metastatic LM7 OS cell line when injected intravenously (Fig. 3C,D). Such effects on lesion size implied that WWOX affects metastatic colonization (Fig. 3) in addition to its influences on local invasion events (Fig. 2). These data are also consistent with WWOX effect on primary tumor growth [(Fig. 2A) and^12^] indicating that WWOX could affect cell growth as well as invasion.

### WWOX restoration in OS cell lines is associated with decreased levels of RUNX2 target genes involved in cell adhesion and motility

**Figure 4:**
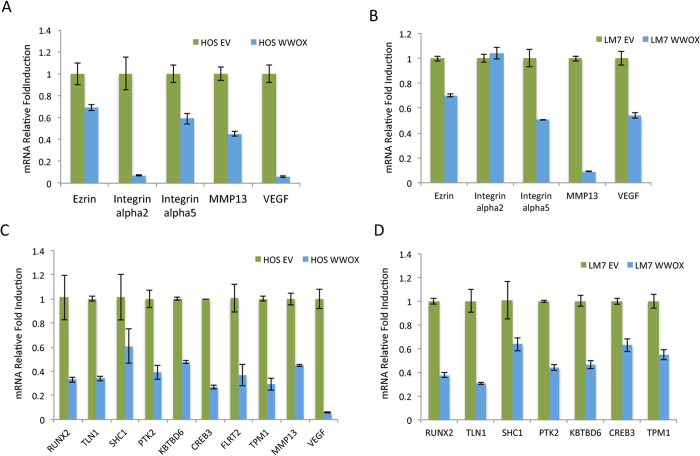
High levels of WWOX in OS cells are associated with decreased expression of RUNX2 target genes. (**A,B**) HOS and LM7 control and WWOX-restored cells were subjected to real-time PCR for genes known to be involved in OS metastasis. (**C,D**) RUNX2 target genes are significantly downregulated upon WWOX restoration in both HOS and LM7 cell lines. Relative fold induction is shown. Results are expressed as mean ± SEM. *p < 0.05 as compared with HOS/LM7-EV cells.

Our results so far indicate that WWOX restrains OS metastasis. To shed light on the mechanism by which WWOX could exert its anti-metastatic activity, we perform real-time PCR analysis on known metastatic genes implicated in OS progression. To this end, total RNA was isolated from manipulated HOS and LM-7 cells. Our results indicate that the expression of ezrin, integrins alpha 4 and 5, MMP13 and VEGF were reduced upon WWOX expression in HOS and LM7 cells (Fig. 4A,B).

Since it has been shown that RUNX2 regulates the expression of genes involved in cell motility and adhesion in OS^24^ and WWOX suppresses RUNX2 transactivation^12^,^25^, we set to determine whether WWOX expresssion modulates expression of RUNX2 target genes in OS metastatic cells. As shown in Fig. 4C,D, we found that RUNX2 target genes related to tumor properties are significantly downregulated in HOS-WWOX and LM7-WWOX OS cell lines when compared to the EV cells. In conclusion our findings indicate that *WWOX* overexpression is accompanied by the downregulation of genes that are involved in tumor cell migration and invasion rendering them less metastatic and less aggressive.

## Discussion

Several studies have demonstrated that WWOX inactivation is associated with a more aggressive tumor behavior in different cancers^10^,^26^. Our previous data and others showed that WWOX expression is absent or reduced in the majority of OS^12^,^13^,^14^,^27^ and that restoring WWOX levels in OS cell lines results in inhibition of tumorigenicity^12^. Here we demonstrated, for the first time, that WWOX also attenuates metastatic potential of OS cells. Indeed, our findings show that WWOX restoration in HOS and LM7 cell lines can attenuate their metastatic behavior both *in vitro* and *in vivo*, likely through regulation of RUNX2 levels and activity.

The data presented here shed light on pleiotropic functions of WWOX in suppressing tumoriogenesis. Ectopic expression of WWOX has been shown constantly to suppress primary tumor growth of different cancer types not only in OS^12^ but also in carcinomas of the lung^28^, breast^29^, prostate^30^ and pancreas^31^. Our findings now further show that WWOX could affect metastasis. Metastasis is a multistep process that requires the upregulation of several molecules that have a role in inducing angiogenesis such as VEGF; facilitating the degradation of the ECM like upregulation of certain MMPs; surviving and driving the homing to distant organs including upregulation of ezrin, selectins and integrins^6^. Many of these genes were found to have a peculiar role in OS metastasis^6^. Importantly, our data show that expression of WWOX suppresses the expression of many of these genes and pathways to suppress seeding and survival of metastatic cells.

Another finding that emerge from our study is that WWOX suppresses RUNX2 transactivation function of target genes implicated in migration and adhesion in OS cells. RUNX2 is the principle regulator of osteoblast differentiation and has been shown to be upregulated in OS^12^,^27^,^32^,^33^. We previously showed that RUNX2 is expressed in the majority of primary tumors and is undetectable in most tumors resected following chemotherapy, whereas most metastases were RUNX2 positive^12^. RUNX2 levels are also elevated in in femurs of *Wwox*-deficient mice^12^. Furthermore, expression of WWOX in OS cells is associated with reduced RUNX2 levels and RUNX2-target genes^25^. These data promoted us to examine the effect of WWOX expression in metastatic OS cells on RUNX2 target genes. We found that WWOX reconstitution in HOS and LM7 cells is associated with downregulation of RUNX2 levels. Although RUNX2 is a natural suppressor of normal osteoblast proliferation, it is aberrantly expressed at elevated levels in a subset of cells derived from patients with OS. Its expression significantly correlated with metastasis and predicted a trend towards lower survival^33^. An extensive but incomplete catalog of RUNX2 target genes expressed in osteoblasts, as well as in OS and other non-osseous tumors has emerged 32,34,35,36. These genes generally alter pathways linked to cell proliferation and survival, as well as other cellular activities required for tumorigenesis or cancer metastasis. In fact, RUNX2 activates genes necessary for angiogenesis (VEGF, osteopontin)^37^, metastasis and invasion (MMP2, MMP9, MMP13)^38^,^39^ and survival (survivin)^40^. Van der Deen *et al.* have recently analyzed the genomic function of RUNX2 in OS cells to gain insight into molecular pathways that are perturbed in bone cancer. Their results showed that RUNX2 controls pathways that are broadly related to cell adhesion and motility and renders OS cells more aggressive^24^. When levels of these genes in WWOX manipulated OS cells were examined, we found that WWOX overexpression represses levels of most of these genes. These results further indicate that WWOX loss might contribute to OS progression by inhibiting several genes implicated in metastasis through regulation of RUNX2. The role of RUNX2 in tumor progression of other tumors including breast and prostate has been revealed^38^,^41^,^42^,^43^; therefore it is plausible to assume that WWOX expression could also attenuates metastasis of these neoplasms. The modulation of RUNX2 pro-metastatic activity by WWOX could also be important for other RUNX2-related functions like osteoblast differentiation and ribosomal biogenesis^44^.

Emergence evidence is associating WWOX function with metabolism and genome stability. Depending on timing of WWOX loss and context of what other tumor suppressors or oncogenes are modulated, the functional outcome of WWOX is shaped. For example, WWOX could be lost during early stage of carcinogenesis impairing DNA damage response and hence promoting genome instability and cancer evolution^8^. WWOX loss could also be significant during tumor progression modulating gene expression thus leading to tumor promotion and metastasis. Role of tumor suppressor WWOX in metastasis is poorly explored, though some studies suggested a potential role. For example, Gourley *et al.* suggested that loss of WWOX contributes to the peritoneal dissemination of human ovarian cancer cells via modulating the interaction between tumor cells and the extracellular matrix^45^. Consistent with this notion, knockdown of WWOX in MCF10A is associated with increased levels of fibronectin^46^. In gastric signet-ring cell carcinoma, WWOX was shown to regulate activity of TMEM207 to suppress invasion and metastasis^47^. Nevertheless, non of these studies directly addressed and followed role of WWOX in metastasis. Through modulating WWOX levels in WWOX-negative OS cells, we now showed that WWOX attenuates invasion potential of OS cells in primary site (tibia) as well as their colonization in secondary site (lung). Whether WWOX affects metastasis of other cancer cell types is very likely but this has to be addressed in future studies.

Another emerging function of WWOX is maintaining homeostasis of the nervous system. Modeling loss of WWOX in animal models is associated with epilepsy and ataxia^18^,^48^. In agreement with these observations, several recent reports have described that germline homozygous mutations of the *WWOX* gene in human patients are associated with epileptic encephalopathies and early death, in most patients, precluding adult tumor analysis^18^,^49^,^50^,^51^. It remains unknown whether patients with missense mutations are more sensitive to tumor development, including OS, during their life span. A genetic variant of the *WWOX* locus (CNV-67048), which is associated with reduced WWOX expression, has been recently correlated with predisposition to lung cancer and gliomas^52^,^53^,^54^. These results suggest that WWOX posses pleiotropic functions affecting metabolism, nervous system as well as cancer development^55^,^56^,^57^. The prevalence of one phenotype over the other could likely be related to context (e.g. tissue type or occurrence of other mutations) though more studies should be required to better understand the role of WWOX in biology and cancer.

In conclusion, our data suggest that WWOX expression, which is commonly lost or reduced in a large portion of human OS, could suppress OS pulmonary metastasis likely through regulation of RUNX2 function.

## Additional Information

**How to cite this article**: Del Mare, S. and Aqeilan, R. I. Tumor Suppressor WWOX inhibits osteosarcoma metastasis by modulating RUNX2 function. *Sci. Rep.* **5**, 12959; doi: 10.1038/srep12959 (2015).

## Figures and Tables

**Table 1:** Primer sequence for qRT-PCR analysis.

Gene	Forward Primer	Reverse Primer
*hWWOX*	5′-TCCTCAGAGTCCCATCGATTT-3′	5′-CGGCAGCAGTTGTTGAAGTA-3′
*hGAPDH*	5′-A TGGGGAAGGTGAAGGTCGG-3′	5′-TGACGGTGCCA TGGAA TTTG-3′
*hRUNX2*	5′-ACCAGATGGGACTGTGGTTAC-3′	5′- CGTTGAACCTTGCTACTTGGT-3′
*hMMP9*	5′-TCTTCCCTGGAGACCTGAGA-3′	5′-AGGGTGGGTAGTCATTTGCATAG-3′
*hCOL1A1*	5′-GGTGTTGTGCGA TGACGTGA-3′	5′-TCATAGCCATAAGACAGCTG-3′
*hTLN1*	5′- CAACCAGCTAGATGAAGGAC-3′	5′- TTGACTTGGTAACCATCTCCT-3′
*hSHC1*	5′- GAGCTACATTGCCTGTAGGA-3′	5′- TCTAGGTTCTGGACGTTGAC-3′
*hPTK2*	5′- TATATGAGTCCAGAGAATCCAG-3′	5′- GCTTCACAATATGAGGATGGT3′
*hKBTBD6*	5′- TCTCTGCTCGTGTTTATCCT-3′	5′- ATCCACCTAAGTCCCATTCAG-3′
*hCREB3*	5′- AGACACTTCCTCTCACTAAGAC-3′	5′- GTATTTCAAGACCCTGCTCTC-3′
*hFLRT2*	5′- CCGTCAGATTGCAGATTGAG-3′	5′- CAGGTGATGGCAAGTTATCAG-3′
*hTPM1*	5′- GATGACTTAGAAGAGAAAGTGG-3′	5′- TAACTCCAGTAAAGTCTGATCC-3′
*hMMP13*	5′- ATGAGCCAGAGTGTCGGTTC-3′	5′- GTTAGTAGCGACGAGCAGGAC-3′
*hVEGF*	5′- GAGGAGCAGTTACGGTCTGTG-3′	5′- TCCTTTCCTTAGCTGACACTTGT-3′
*hEZRIN*	5′- ACCAATCAATGTCCGAGTTACC-3′	5′- GCCGATAGTCTTTACCACCTGA-3′
